# Item bias on the geriatric depression scale (GDS): investigating the quality and generalizability of GDS on Chinese and Korean community-dwelling elderly population

**DOI:** 10.1186/s12877-021-02516-z

**Published:** 2021-11-09

**Authors:** In Hye Park, Yustika Sya’bandari, Yang Liu

**Affiliations:** 1 School of Nursing, University of Washington-Seattle, Seattle, USA; 2 Visi Prima Nusantara, North Jakarta, Indonesia; 3 Nursing Department, School of medicine, Xiamen University, Xiamen, China

**Keywords:** Chinese, Differential Item Functioning, Generalizability, Geriatric Depression Scale, Korean

## Abstract

**Background:**

Although some previous studies have reported the impact of cultural factors on individuals’ cognition and decision making, a shortage of research has led to this comparison study for Chinese and Korean elderly, a growing population with depression. This study aimed to explore depression levels in Chinese and South Korean elderly individuals by focusing on testing the generalizability of the Geriatric Depression Scale (GDS).

**Methods:**

The data of 493 community-dwelling Chinese and Korean elderly individuals over the age of 60 years were used to examine GDS. To test the dimensionality, item quality, and reliability of the GDS, the item response theory, Rasch analysis was performed. The detection of differential item functioning (DIF) of the GDS between the two countries was determined by performing a hybrid ordinal logistic regression.

**Results:**

The four-dimensional framework of the GDS, categorized into agitation, cognitive concerns, dysphoria, and vigor/withdrawal was fit for measuring depression levels in Chinese and Korean elderly individuals. In addition, good item quality and reliability of the GDS indicate that almost all items in this scale contribute to measuring the intended trait. Meanwhile, 18 out of 28 items of the GDS were detected as country-related DIF with five items having a large effect size.

**Conclusions:**

Although China and Korea are close geographically and culturally, the item bias shown by severe country-related DIF implies that different cultural backgrounds impact how the elderly interpret GDS items. The cultural issues related to the specific DIF items, the implication to accuracy of individual scores estimation, and the optimal decision to treat individuals were discussed.

**Supplementary Information:**

The online version contains supplementary material available at 10.1186/s12877-021-02516-z.

## Background

Depression is a common psychiatric disorder in elderly individuals [1]. Although its prevalence differs based on the diagnostic criteria, it is reported to range from 4.5 to 36 %, including subthreshold depressive symptoms [2]. The main attributions of depression in the elderly population include decreased physical functioning, aging-associated diseases, reduced social roles due to retirement, decreased economic status, and isolation or loneliness from the loss of loved ones [1, 3, 4]. In previous research, negative health consequences associated with depression have shown an increased risk of physical and cognitive dysfunction, dementia, cardiovascular and neurologic disorders, suicidal ideation from social isolation, and finally mortality [3, 5]. Additionally, a positive association between increased medical expenses, depression screening, and related treatments has been reported [6–8]. Decreased physical and psychological health, social burden caused by depression, and its management worsen the symptoms leading to a lower quality of life [9–11]. Considering the current situation of a rapidly increasing aging population [12–14], accurate diagnosis of depression and effective treatment planning based on the assessment are essential.

Several self-reported measures assess depression, but the geriatric depression scale (GDS) is one of the most widely used screening tools for the elderly [1, 15]. However, Barua et al. reported that the prevalence of depression in the elderly population varied between 10 % and 20 % worldwide, suggesting that cultural issues are critical considering the challenge of cultural translation regarding the definition of depression in culturally diverse groups [16]. About this issue, Matsumoto argued that cultural background impacts the formation of diverse personal feelings and influences the way people express distress [17]. Additionally, the process of inferential thinking and decision-making may result from comparing themselves with their cultural group, called a reference group effect [18, 19]. It is because culture determines how we assume about, recognize, and interpret the health [20]. In other words, although people have similar symptoms, the reported scores might be different due to the conflation of cultural factors in one’s cognition. Indeed, this phenomenon has been described in previous studies showing different levels of responses to specific psychological tests [20]; nonetheless, Im et al. suggested some additional criteria for the evaluation of cross-cultural studies considering different cultural aspects [21]. It is significance to be aware about culture’s effect on health practice. Given that people from various cultural backgrounds seeking health care facilities undergo psychological tests often to assess their condition at their first visit and follow-up, the responses from self-reported measurements should be comprehensively validated to obtain more accurate data interpretation.

Messick proposed six aspects of construct validity of psychological assessments: content, substantive, structural, generalizability, external, and consequential [22]. These aspects take into consideration the interpretation of the score meaning as well as the consequences of score use. A crucial aspect related to instrument performance to measure respondent trait across different groups was to test generalizability. Particularly, in an international comparison study, the instrument expected to measure the intended trait in the same manner across different groups of participants (e.g., ethnicity, gender, religion) [23]. To test the generalizability of the instrument, differential item functioning (DIF) analyses can be performed. It detects biased items as they may be easier or difficult for a particular group [24]. An instrument with no DIF refers to the standard of measurement, assessing performance without favoring certain individuals [24]. Testing the DIF is fundamental in test development and evaluation.

Previous research reported East Asia countries have culturally interdependent self-construal which possibly affect the increasing stress level [25]. China and Korea have become representative East Asian countries of an aged society that are expected to account for over 30 % of people aged over 60 in the near future [26]. Furthermore, the number of older people with depression or depressive symptoms has been growing in both countries [27, 28]. Depression’s prevalence, assessment, and treatment have been closely linked to sociocultural, economic, and political aspects [29–31]. Hence, this study focuses on China and Korea society.

China and Korea show the approval of traditional Confucian values in cultural contexts [32]. Despite sharing common history and culture to a particular degree, both countries potentially exhibit different cultural and social responses. Hofstede cultural dimensions theory well presents the influence of culture on societal value and behavior. According to the findings, the levels of masculinity and uncertainty avoidance in China and South Korea are substantially different (see: www.hofstede-insights.com). China is estimated having higher masculinity, which reflects a more competitive culture where society motivated to be the best in achieving success. Uncertainty avoidance, on the other hand, Korea is estimated showing higher feeling of threatened by ambiguity circumstances, which is linked to how society deals with anxiety. As the East Asian countries, China and Korea included collectivist countries which might prefer to share stressful issues with others. Nonetheless, China has slightly higher levels of individualism. Besides, a cross-cultural comparison study of East Asian countries mentioned that China presents a higher value interpersonal harmony [32], where people have to discipline and express the emotions in diverse relationship to achieve the harmony [32, 33]. Furthermore, China also demonstrated higher tradition and social hierarchy (rules, status, and authorities) [32], and it is accordance to Hofstede’s dimension that China shows higher score of power distance where the culture accepts the inequalities among individuals.

To challenge the assumption of East Asian societies which is viewed to implement the culture and values similarly, this study examines how the different background of society impacts on individual responses of the depression items. Particularly, it explores the validation of GDS focused on testing generalizability of the instrument. The validation for GDS in this international comparison study will help promote cultural knowledge and sensitivity needed from clinical practitioners and researchers, and the results of this study can ultimately be used as the basis for depression assessment and interpretation. Specifically, this study validates the response of individual depression levels measured by the GDS across Chinese and Korean community-dwelling elderly populations with the following research questions:

1. Does the GDS have the best unidimensional or multidimensional frameworks?
2. How well do the GDS items measure traits within underlying constructs?
3. Are the GDS items generalizable across Chinese and Korean participants?

## Method

### Study design and participants

This is a cross-sectional study design. For the recruitment of elderly individuals, convenience sampling between October 2016 and October 2017 was used. Particularly, data were collected from participants who resided in the two districts of Daejeon city in South Korea and four districts of Xiamen city in China. All participants fulfilled the following requirements: (1) at least 60 years of age, (2) no cognitive impairment, and (3) verbal communication competency. A total of 527 community-dwelling elderly (297 Chinese and 230 Korean) were recruited and 493 people out of them were finally responded (93.5 %) and investigated. The participants were provided written consent to participate in the study, and were guaranteed anonymity and confidentiality. All methods were performed in accordance with the relevant guidelines and regulations by including a statement in the ethics approval and consent to participate section. All data were obtained through interviews by trained personnel (investigators in this study, senior undergraduate, and graduate students in nursing). They were trained to fill in the Chinese and Korean versions of GDS before starting the data collection in order to verify the authenticity and reliability of the results. When the survey was completed, the questionnaires were checked one by one to verify missing items or ambiguity. The elderly who participated in the survey were given toothbrushes as a token of appreciation. In Table 1, the participants’ characteristics were presented.

**Table 1:** The characteristics of participants

Country		China	Korea
Number of participants		287 (58.2 %)	206 (41.8 %)
Age (*t* = -8.18, *p* = < 0.001)		72.3 (SD = 7.6)	78.1 (SD = 7.8)
Gender	Male	149 (51.9 %)	62 (30.1 %)
	Female	138 (48.1 %)	144 (69.9 %)
Marital Status	Without Partner	101 (35 %)	131 (63.6 %)
	With Partner	186 (64.8 %)	75 (36.4 %)
Lifestyle	Living Alone	59 (20.6 %)	52 (25.2 %)
	Living with Family	228 (79.4 %)	151 (73.3 %)
Education	No Education	66 (23 %)	67 (32.5 %)
	Elementary School	97 (33.8 %)	72 (35 %)
	Middle School	63 (22 %)	16 (7.8 %)
	High School	41 (14.3 %)	31 (15 %)
	Community College	10 (3.5 %)	2 (1.0 %)
	Bachelor/Graduate	10 (3.5 %)	18 (8.7 %)
Job before age 60	Not Employed	32 (11.1 %)	69 (33.5 %)
	Employed	253 (88.2 %)	137 (66.5 %)
Social activity (None 1, Sometimes 2, Often 3) (z = -5.766, *p* = < 0.001)		1.88 (SD = 0.72)	2.27 (SD = 0.72)
Perceived health (Very poor 1, Poor 2, Average 3, Good 4, Excellent 5) (z = -1.132, *p* = 0.258)		3.18 (SD = 1.08)	3.08 (SD = 0.89)
Perceived economic status (Low 1, Middle-Low 2, Middle 3, Middle-High 4, High 5) (z = -3.756, *p* = < 0.001)		2.54 (SD = 0.99)	2.84 (SD = 0.77)
Perceived being caring (Very poor 1, Poor 2, Average 3, Good 4, Excellent 5) (z = -5.388, *p* = < 0.001)		3.79 (SD = 0.87)	4.20 (SD = 0.74)

### Instrument

The Geriatric Depression Scale (GDS), originally developed by Yesavage et al. in 1982, is a self-report instrument used extensively for comprehensive geriatric assessment of depression [34]. It is a close-ended, yes/no response questionnaire comprising 30 items in one-dimensional frameworks. Excluding somatic symptoms, the GDS asks participants about their interest, satisfaction, and worries about life and social activities over the past week. Since the data were collected from Chinese and Korean elderly samples, the Chinese [35, 36] and Korean [37] versions of the GDS were administered for this study. A higher GDS score indicating a higher perception of depression (score range, 0–30). The reliability of Chinese version of GDS was 0.85 (Cronbach’s alpha) and 0.83 (split-half reliability), and 0.81 (test-retest reliability), respectively [36]. Meanwhile, the Cronbach’s alpha and Split-half reliability of Korean version of GDS were 0.88 and 0.79, respectively [37]. Specifically, the correlation value with GDS was 0.87. In addition, KGDS revealed five dimensions with a variance of 53.72 % [37].

### Data analyses

#### Dimensionality, item quality, and reliability

To test the quality of the GDS, two analyses were conducted as follows. First, the dimensionality test was intended to assemble the items coherent with each other and construct the framework of the instrument [38]. We performed item response theory (IRT)-Rasch analyses using ConQuest version 4.14.2. The original GDS with a unidimensionality framework [34], was compared with multidimensional frameworks [39–46]; the framework with a lower deviance and Akaike information criterion (AIC) value indicated a better model [47–49]. This study tested these values on both Chinese and Korean participants, as well as on all participants simultaneously. Second, item quality was tested by investigating the item fit (infit and outfit mean square values [MNSQ]). Item fit explains the item’s function in measuring the trait being measured. The prevalent benchmark to determine the productive measurement is 0.5–1.5. Additionally, the suggested acceptable value is within 0.5–1.7, for clinical observation [48, 50]. The average MNSQ value at 1 means that the estimated model and the data being observed are fitted [48].

With regards to reliability of GDS, the value was explored by analyzing the expected a posteriori/plausible value (EAP/PV) reliability and person separation reliability [51]. The EAP/PV reliability measures the consistency of a set of item difficulties when tested in different respondents with similar abilities [38]. The person separation reliability was comparable to evaluating internal consistency [50, 52], and assumed to be similar to reliability indices such as Cronbach’s alpha. Finally, Cronbach’s alpha of the classical test theory (CTT), broadly used in many studies, was examined to complement the results of the IRT. The reliability value is bounded by 0 and 1, and the closer it is to 1, the less variability of the measurement error [48]. Nevertheless, the value of reliability more than 0.7, includes acceptable consistency [53, 54]. Fischer recommended the following specific categories for the reliability value: excellent (> 0.94), very good (0.91 to 0.94), good (0.81 to 0.91), fair (0.67 to 0.80), and poor (< 0.67) [55].

#### Non-parametric and parametric DIF: magnitude and impact

The generalizability of the GDS items in the two countries was examined by testing differential item functioning (DIF). DIF detects whether participants with the same traits, coming from different subgroups interpret the item differently [23, 50]. The non-parametric DIF test has been broadly used because it is considered to provide insight into the potential DIF [56]. The Mantel-Haenszel test was performed in this study. A previous study argued that this can only detect non-uniform DIF. However, the non-parametric test is still used by many researchers because it is easily used and understood by readers, and does not require the specification of a model to describe the relationship between item performance and the group variable [57]. The DIF is exhibited if the *p-*value is less than 0.05 [57]. Researchers employed IRT for DIF detection owing to theoretical and practical considerations in applications [56]. In this study, the parametric DIF was analyzed by performing a hybrid ordinal logistic regression provided by the lordif package [58]. This was considered an advanced DIF analysis because it can detect the type and magnitude of the DIF. By performing lordif, each item forms the null and three nested models with additional explanatory variables [58].

The detection and magnitude of DIF were determined by comparing the probability (*χ*^2^) and Nagelkerke effect size values (*R*^2^). First, the significant ratio of the likelihood value between models 1 and 2 (*df* = 1) detected a uniform DIF. The uniform DIF indicated that the effect of DIF was constant for all participants at different levels of the trait. Second, the significant ratio of the likelihood value between models 2 and 3 (*df* = 1) detected the non-uniform DIF; it refers to the various effects of DIF, which depend on the level of traits. Lastly, the significant ratio of likelihood value between models 1 and 3 (*df* = 2) indicated the overall DIF/total DIF (uniform and non-uniform). We used the predetermined cut-off of the significant probability value of DIF detection (< 0.01) [58]. Additionally, the magnitude of DIF was determined by the *R*^2^ value model comparison. According to Gelin and Zumbo [59], the benchmark is categorized into negligible effect size (< 0.035), moderate effect size (0.035–0.07), and large effect size (> 0.07). Furthermore, the lordif calculated *Δβ*_1_ for each item complementing the *R*^2^ values to interpret the effect size of the DIF, indicating a meaningful DIF effect if the value is > 0.05.

The lordif produces plots depicting the effect of accumulated DIF on the group and individual scores. The impact of DIF on individual scores can be determined by examining the change in theta estimate with and without the adjustment of DIF or subtraction of purity from the initial theta [58]. The initial theta (unadjusted theta) was generated from the model by accounting for the DIF, and all parameters were set equally for both the groups. Meanwhile, the purified theta (adjusted theta) refers to the score excluding the DIF or the score generated from the model that is estimated separately from the groups [60]. Additionally, both scores were compared using an independent sample t-test to identify significant differences in the scores before and after adjustment.

## Results

### RQ1. The frameworks of GDS

Table 2 presents the results of the dimensionality test of the GDS based on different frameworks proposed by previous studies. The four-dimensional framework suggested by Haavisto and Boron [42], was the best fit for Chinese elderly population, showing low final deviance and AIC values than those of other models (final deviance = 8159.10, AIC = 8235.10). On the other hand, the dimensionality model proposed by Kim et al. [43], showed a greater fit with Korean elderly population (final deviance = 5313.70, AIC = 5401.70). After combining all participants’ data, the four-dimensionality model proposed by Haavisto and Boron [42], was better for GDS (Final deviance = 14028.57, AIC = 14104.57). Hereinafter, observing Korean data in detail, although the five-dimensional model by Kim et al. [43], showed lower final deviance and AIC, these values were not considered to be significantly different from the four-dimensional model given by Haavisto and Boron [42]. Thus, both frameworks can be assumed to fit Korean elderly individuals. Finally, this study decided on a four-dimensional model for further analyses of the GDS. The four dimensions include agitation, cognitive concerns, dysphoria, and vigor/withdrawal.

**Table 2:** The dimensionality test of GDS on Chinese and Korean elderly

Model	Criterion	China	Korea	All
One-dimension Yesavage et al. [34]	Final Deviance	8922.03	5820.61	15201.88
	AIC	8984.03	5882.61	15263.88
Two-dimension Ertan and Eker [39]	Final Deviance	8889.48	5701.98	15066.04
	AIC	8955.48	5767.98	15132.04
Three-dimension Pocinho et al. [45]	Final Deviance	8658.92	5573.19	14723.43
	AIC	8730.92	5645.19	14795.43
Four dimension Haavisto and Boron [42]	Final Deviance	8159.10	5399.41	14028.57
	AIC	8235.10	5475.41	14104.57
Four-dimension Ganguli et al. [41]	Final Deviance	8889.05	5709.23	15052.39
	AIC	8969.05	5789.23	15132.39
Five-dimension Galeoto et al. [40]	Final Deviance	8840.74	5667.89	15006.81
	AIC	8930.74	5757.89	15096.81
Five-dimension Kim et al. [43]	Final Deviance	8633.65	5313.70	14155.39
	AIC	8723.65	5401.70	14243.39
Five-dimension Sheikh et al. [46]	Final Deviance	8868.15	5661.04	15037.96
	AIC	8958.15	5751.04	15127.96
Six-dimension Parmelee, Lawton, and Katz [44]	Final Deviance	8657.47	5603.35	14782.20
	AIC	8759.47	5705.35	14884.20

### RQ2. Item quality and reliability of GDS

Haavisto and Boron [42], mentioned that the items GDS7 and GDS9 were removed from the scale because they comprised only two items in each dimension. This could not substantiate the underlying latent factors for conceptual relevance. Thus, only 28 GDS items were used within the four-dimensional framework. The results of item quality testing revealed that almost all items conformed to a good fit with the Rasch model, except for GDS23 in dysphoria. Table 3 demonstrated the MNSQ range of agitation (infit = 0.92–1.19, outfit = 0.78–1.31), cognitive concerns (infit = 0.96–1.11, outfit = 0.94–1.18), and vigor/withdrawal (infit = 0.83–1.27, outfit = 0.56–1.35). These values are within the cut-off range of 0.5–1.7 for clinical observation. Nonetheless, the range of dysphoria was 0.82–1.46 for infit and 0.66–2.01 for outfit MNSQ. The GDS23 was a misfit because of its outfit MNSQ’s outlier value.

**Table 3:** The item quality of GDS in four dimensions

Dimension	Item	Estimate	Infit MNSQ	Outfit MNSQ	Cronbach’s alpha if Item deleted
Dimension 1: Agitation	GDS6	-0.072	1.05	1.07	0.669
	GDS8	-0.172	0.95	0.87	0.570
	GDS13	-0.021	0.94	0.85	0.569
	GDS18	0.730	0.92	0.78	0.584
	GDS29	-0.464	1.19	1.31	0.684
Dimension 2: Cognitive concerns	GDS14	-0.468	1.11	1.18	0.490
	GDS26	-0.004	1.01	0.95	0.538
	GDS30	0.472	0.96	0.94	0.387
Dimension 3: Dysphoria	GDS1	0.671	0.88	0.72	0.824
	GDS2	-1.463	1.22	1.48	0.843
	GDS3	-0.491	0.93	0.93	0.824
	GDS4	-0.374	0.82	0.66	0.815
	GDS10	0.031	0.93	0.82	0.826
	GDS11	0.583	0.92	0.92	0.825
	GDS16	-0.139	0.84	0.71	0.819
	GDS23	-0.503	1.46	2.01	0.855
	GDS24	0.511	0.97	0.78	0.829
	GDS25	1.173	0.96	0.85	0.831
Dimension 4: Vigor/withdrawal	GDS5	-0.932	1.00	0.96	0.764
	GDS12	0.054	1.27	1.35	0.786
	GDS15	0.306	1.02	1.13	0.771
	GDS17	0.948	0.88	0.67	0.760
	GDS19	-1.034	0.93	0.91	0.754
	GDS20	-1.828	1.09	1.23	0.779
	GDS21	-0.735	0.84	0.73	0.743
	GDS22	1.405	0.83	0.56	0.761
	GDS27	0.823	1.01	1.01	0.772
	GDS28	0.994	1.19	1.32	0.779

As shown in Table 4, for the reliability values, the results of the person separation reliability of the Chinese, Korean, and combined data were 0.980, 0.981, and 0.987, respectively. It was considered acceptable because the values were more than 0.7; particularly, they were categorized as having excellent reliability based on Fischer’s [55]. Additionally, the results of EAP/PV reliability presented acceptable values (ranging from fair to good), except for the cognitive concerns dimension; the values of Chinese participants and combined data were 0.659 and 0.639 (poor). Furthermore, Cronbach’s alpha analysis demonstrated low reliability values for the dimensions of agitation and cognitive concerns, whereas demonstrated good values for dysphoria and vigor/withdrawal dimensions.

**Table 4:** The reliability of GDS in four dimensions

Data	IRT-Reliability					Cronbach's Alpha			
	EAP/PV Reliability				Person Separation Reliability				
	D1	D2	D3	D4		D1	D2	D3	D4
Chinese	0.705	0.659	0.821	0.815	0.980	0.667	0.600	0.801	0.785
Korean	0.784	0.702	0.883	0.849	0.981	0.713	0.597	0.884	0.803
All	0.719	0.639	0.850	0.823	0.987	0.669	0.573	0.844	0.786

### RQ 3. Generalizability of GDS items on Chinese and Korean elderly

As presented in Table 5, the results of DIF detection demonstrate that several GDS items were not generalized to the groups of Chinese and Korean participants. Non-parametric analyses revealed 18 DIFs out of 28 GDS items (*p* < 0.05). Particularly, there were three items each for agitation and cognitive concerns, and six items each for dysphoria and vigor/withdrawal dimension. Additionally, a total of 18 items were flagged as DIF in the parametric analysis (*p-value* = < 0.01). Three DIF each, were detected in agitation, and cognitive concerns, four in dysphoria, and six in vigor/withdrawal dimension. Among the 18 DIF items, nine items were categorized as uniform DIF (GDS13, GDS29, GDS2, GDS10, GDS23, GDS5, GDS12, GDS20, GDS21), five items as non-uniform DIF (GDS26, GDS25, GDS15, GDS17, GDS19), and four items as overall DIF (GDS6, GDS14, GDS20, GDS28). Most of them were subsets with DIF detected in the non-parametric analysis. GDS3, GDS4, and GDS24 were identified as DIF in the non-parametric analysis, but not in the parametric analysis. Meanwhile, GDS25, GDS15, GDS17, and GDS19 were detected in parametric analysis but not in non-parametric analysis. The details of the detected DIF of the GDS are summarized in the Additional file 1.

**Table 5:** The result of DIF detection based on parametric and non-parametric tests

Dimension	Item	Hybrid IRT- Logistic Regression			Mantel-Haenszel	
		${x}_{12}^2$	${x}_{13}^2$	${x}_{23}^2$	*χ*^2^	*p-*value
Dimension 1: Agitation	GDS6	0.000	0.000	0.001	68.39	0.000
	GDS8	0.979	0.917	0.677	1.20	0.274
	GDS13	0.000	0.000	0.106	21.43	0.000
	GDS18	0.675	0.762	0.544	1.60	0.206
	GDS29	0.000	0.000	0.334	41.26	0.000
Dimension 2: Cognitive concerns	GDS14	0.000	0.000	0.000	88.91	0.000
	GDS26	0.016	0.000	0.000	7.87	0.005
	GDS30	0.715	0.799	0.574	0.97	0.324
	GDS20	0.000	0.000	0.001	41.82	0.000
Dimension 3: Dysphoria	GDS1	0.992	0.866	0.592	0.18	0.674
	GDS2	0.000	0.000	0.707	11.32	0.001
	GDS3	0.035	0.087	0.517	12.55	0.000
	GDS4	0.309	0.509	0.574	3.92	0.048
	GDS10	0.002	0.000	0.014	13.57	0.000
	GDS11	0.225	0.479	0.969	0.62	0.432
	GDS16	0.133	0.257	0.498	0.08	0.775
	GDS23	0.000	0.000	0.109	21.23	0.000
	GDS24	0.025	0.026	0.136	6.37	0.012
	GDS25	0.083	0.000	0.000	1.89	0.169
Dimension 4: Vigor/withdrawal	GDS5	0.000	0.000	0.474	29.54	0.000
	GDS12	0.000	0.000	0.750	28.89	0.000
	GDS15	0.631	0.000	0.000	0.21	0.648
	GDS17	0.062	0.000	0.000	2.57	0.109
	GDS19	0.292	0.001	0.000	3.22	0.073
	GDS20	0.000	0.000	0.048	30.28	0.000
	GDS21	0.000	0.000	0.091	6.49	0.011
	GDS22	0.104	0.169	0.339	1.31	0.253
	GDS27	0.219	0.049	0.033	0.73	0.392
	GDS28	0.000	0.000	0.002	13.11	0.000

The parametric analysis revealed the magnitude of the DIF, presented in Table 6. Of the 18 DIF items, five items were categorized into large effect size (GDS6, GDS29, GDS14, GDS20 in cognitive concerns and vigor/withdrawal), nine into moderate effect size (GDS13, GDS26, GDS23, GDS25, GDS5, GDS12, GDS15, GDS17, GDS28), and four into negligible effect size (GDS2, GDS10, GDS19, GDS21). The results of *Δβ*_1_ also presented 11 DIF items (GDS6, GDS13, GDS29, GDS14, GDS20 in cognitive concerns, GDS2, GDS23, GDS5, GDS12, GDS20 in vigor/withdrawal, GDS21), showing a meaningful effect size (value > 0.05).

**Table 6:** The effect size for significantly detected DIF

Dimension	Item	Hybrid IRT-Logistic Regression			
		${R}_{12}^2$	${R}_{13}^2$	${R}_{23}^2$	*Δβ1*
Dimension 1: Agitation	GDS6	0.100	0.119	0.019	0.178
	GDS13	0.053	0.057	0.003	0.145
	GDS29	0.107	0.109	0.002	0.094
Dimension 2: Cognitive concerns	GDS14	0.161	0.183	0.022	0.507
	GDS26	0.009	0.048	0.039	0.023
	GDS20	0.079	0.098	0.019	0.129
Dimension 3: Dysphoria	GDS2	0.032	0.032	0.000	0.074
	GDS10	0.017	0.027	0.010	0.015
	GDS23	0.045	0.051	0.006	0.134
	GDS25	0.007	0.063	0.056	0.040
Dimension 4: Vigor/withdrawal	GDS5	0.069	0.070	0.001	0.148
	GDS12	0.067	0.068	0.000	0.063
	GDS15	0.000	0.036	0.035	0.000
	GDS17	0.007	0.043	0.036	0.019
	GDS19	0.001	0.017	0.016	0.003
	GDS20	0.081	0.089	0.008	0.113
	GDS21	0.026	0.029	0.003	0.223
	GDS28	0.037	0.062	0.025	0.042

**Fig. 1:**
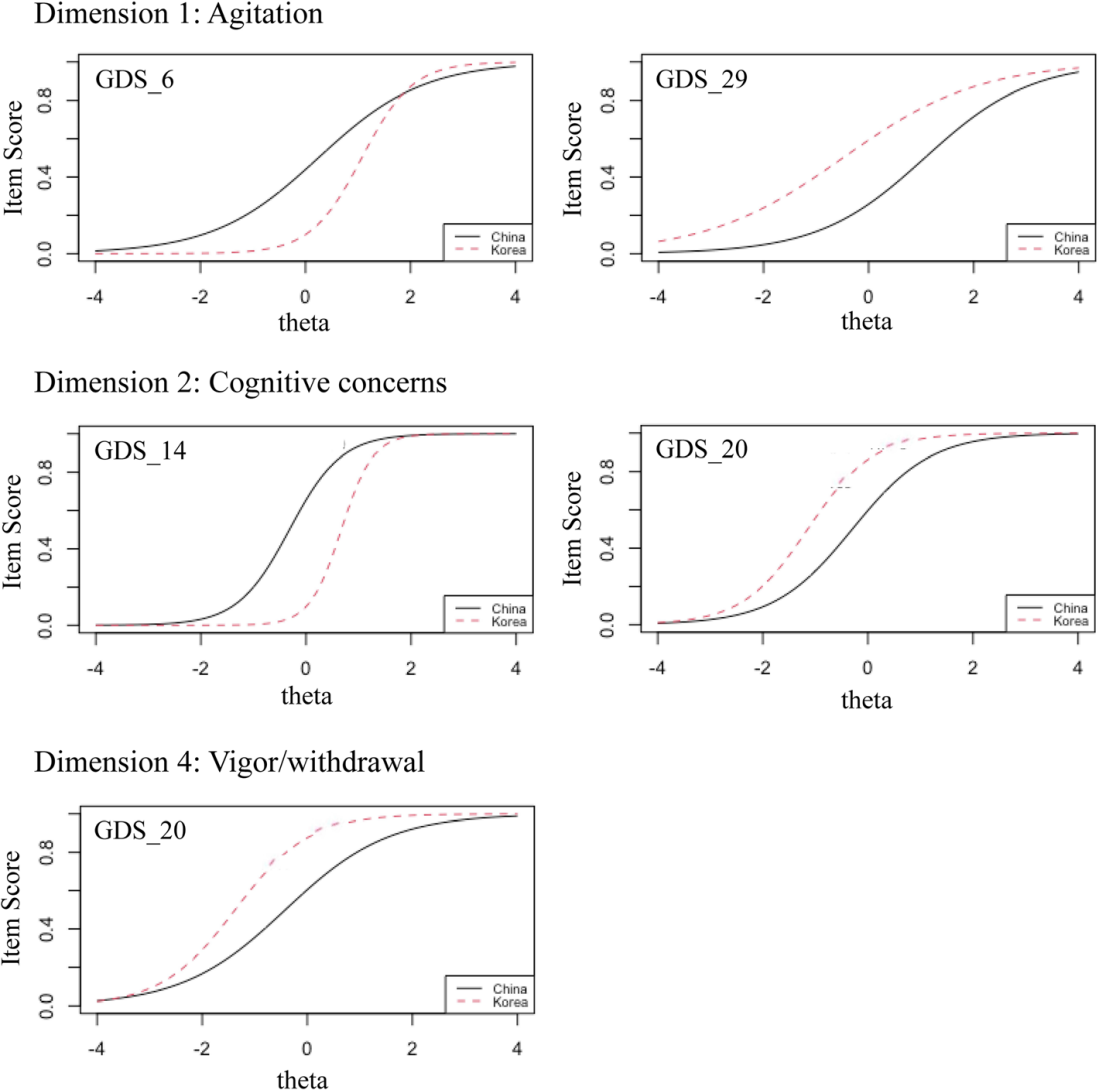
Country-related DIF on GDS with large effect size

Figure 1 depicts the visualization of country-related DIF in the form of item characteristic curves (ICCs). The ICCs illustrate the relationship between the trait (depression level) in the theta unit and the probability of the obtained score (item score). This study presented five ICCs of DIF with a large effect size. A big difference can be seen in the scores obtained by Chinese and Korean individuals at the same level of depression (DIF effect). Particularly, GDS6 shows the total effect of the uniform and non-uniform DIF. At the lower level of depression, a constant effect was seen in which Koreans were expected to score lower. Nonetheless, at a higher level of depression (theta > 2), Koreans were estimated to score slightly higher than the Chinese. Additionally, although the figures demonstrate the constant effect of DIF, GDS14 and GDS20 in the cognitive concerns dimension were also included in both uniform and non-uniform DIF. This might be caused by the slight intersection at the lowest and highest levels of depression. As shown in Fig. 1. GDS14 expected Koreans to score lower than Chinese individuals with the same trait, while Koreans were estimated to score higher on GDS20. Furthermore, GDS29 in agitation and GDS20 in the cognitive concerns dimension presented a uniform DIF, whereas Koreans were expected to have a higher score here than the Chinese participants.

**Fig. 2:**
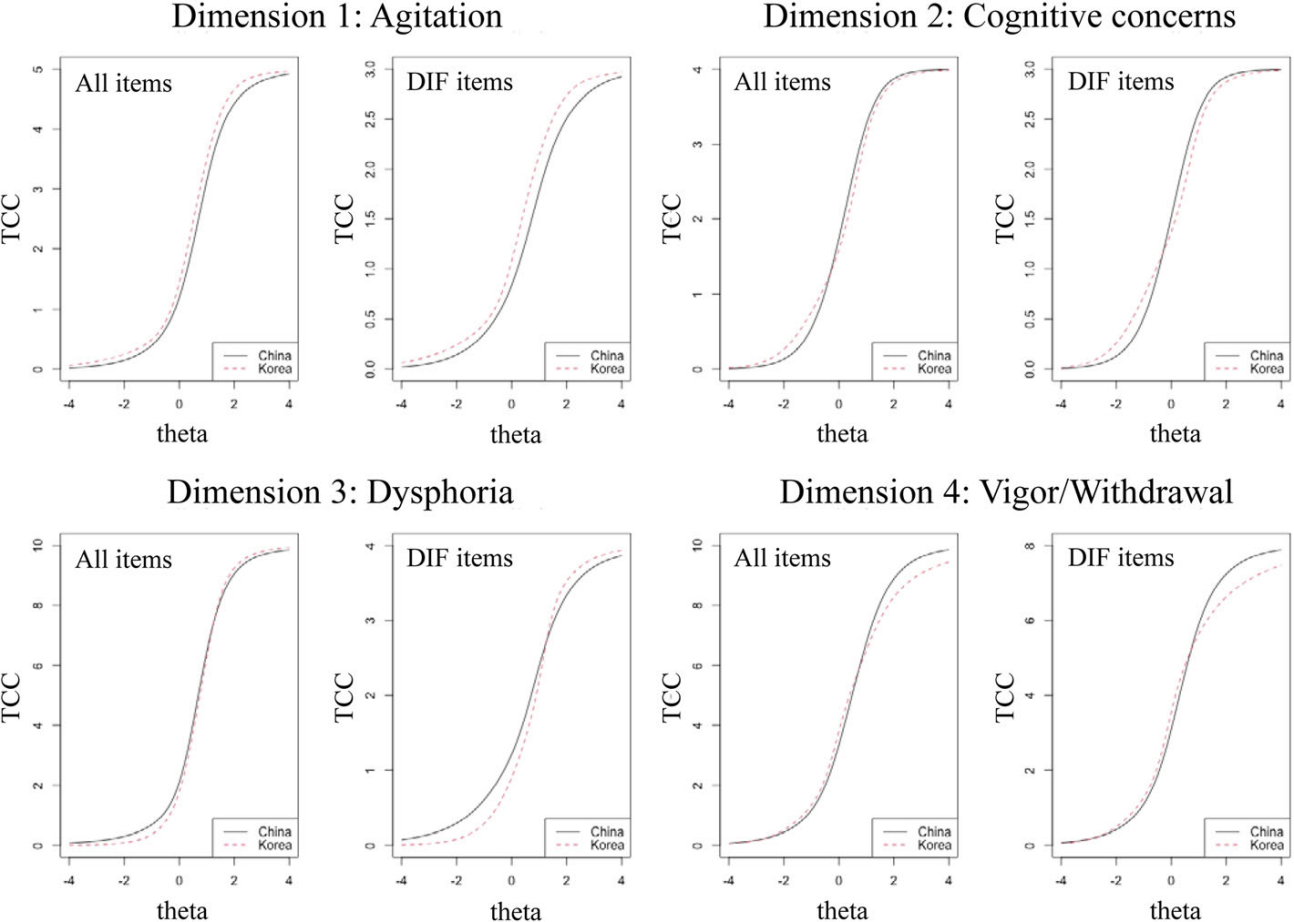
The effect of DIF on test characteristic curves (TCC). The left graph labelled with “All Items” shows the expected total score for the two groups computed from both items with and without DIF. The right graph visualizes the differences between Chinese and Korean elderly in only a subset of items with DIF

Despite each item’s effect on the expected score, the effect of cumulative DIF on the expected score was visualized in the form of a test characteristic curve (TCC). Figure 2 shows the TCC plots for each dimension. The left plot illustrates a small difference in expected scores between the Chinese and Korean participants across different depression levels as it included all item parameters. On the other hand, the right plots show a more obvious difference in the expected score between the two countries because it only accounted for DIF parameters. The difference between the left and right plots in each dimension indicates that the DIF accounted for in the scoring will affect the expected scores. In the agitation dimension, across different levels of depression, Koreans were estimated to have higher scores than the Chinese (uniform). The dimension of cognitive concerns has a different graph because non-uniform DIF is presented. At lower levels of depression (theta − 4 to -1), Koreans were expected to have slightly higher scores, while Koreans estimated to score lower than Chinese at higher levels of depression (theta − 1 to 2). The dysphoria dimension also shows a uniform DIF since, at every level of depression, Koreans are estimated to score lower than Chinese. Nonetheless, at the theta level of more than 2, Koreans expected to score slightly higher. Lastly, the dimension of vigor-withdrawal demonstrates the non-uniform DIF effect, where at lower levels of depression (theta − 3 to 0), Koreans expected to score higher than Chinese, while they estimated to score lower at higher levels of depression (theta 0 to 4).

Furthermore, individual scores before and after adjustment for the DIF effect were obtained. The data of the two countries were compared to reveal that three dimensions, namely agitation, cognitive concerns, and vigor/withdrawal, showed an increase in the *p*-value from the original score to the adjusted score (see Table 7). Particularly, the agitation dimension presented significantly different scores between Chinese and Korean participants in the original scores and a non-significant score when the adjusted scores were compared. This indicates that the expected scores of the two groups were more similar after the adjustment. Additionally, these three dimensions showed a decreasing effect size (Cohen’s *d*) and *t*-value; the dysphoria dimension presented the opposite trend.

**Table 7:** Score comparison between Chinese and Korean elderly before and after adjustment

Dimension	Score comparison	Country	Mean	SD	*t*	*p*	d
Dimension 1: Agitation	Original score	Chinese	-0.070	0.785	-2.295	0.022	0.208
		Korean	0.098	0.830			
	Adjusted score	Chinese	-0.021	0.794	-0.645	0.519	0.059
		Korean	0.027	0.837			
Dimension 2: Cognitive concerns	Original score	Chinese	0.013	0.784	0.457	0.648	0.042
		Korean	-0.019	0.749			
	Adjusted score	Chinese	0.017	0.782	0.043	0.966	0.004
		Korean	0.014	0.792			
Dimension 3: Dysphoria	Original score	Chinese	-0.098	0.795	-2.858	0.004	0.264
		Korean	0.137	0.975			
	Adjusted score	Chinese	-0.116	0.810	-3.331	0.001	0.308
		Korean	0.156	0.950			
Dimension 4: Vigor/withdrawal	Original score	Chinese	-0.028	0.900	-0.815	0.415	0.079
		Korean	0.039	0.884			
	Adjusted score	Chinese	-0.003	0.889	0.273	0.785	0.024
		Korean	-0.025	0.932			

## Discussion

### RQ1. The frameworks of GDS

Before conducting the DIF study, the instrument’s dimensionality framework must be analyzed for the three rationales. First, it must aim to validate the internal structure of the instrument [22] and to assess multiple cognitive dimensions within one assessment [61], ultimately revealing the robust discussion regarding the investigated issue. Second, DIF analysis is performed if the items display good discrimination and show a more homogenous test [62]. It can be presented by the group of items with positive relationships with the total test scores. Accordingly, the items that correlate with each other and measure the same trait should be grouped in the same dimension. Third, as type I measurement errors may occur in a unidimensional analysis [63, 64], the items must be specified based on various measured traits. Nonetheless, previous studies have revealed different multiple frameworks for GDS. Inconsistency in these results may be due to variations in sample characteristics and size. This study revealed that the GDS was consistent with four-dimensional frameworks suggested by Haavisto and Boron [42], including agitation, cognitive concerns, dysphoria, and vigor/withdrawal. First, the agitation dimension comprised five items related to the symptoms of anxiety, asking participants about their worry, fear, or something burdensome in the past and future (e.g., *Do you frequently worry about the future*?). Second, the cognitive concerns dimension comprised three items (e.g., *Do you feel you have more problems with memory than most?*) which are similar to the dimension of cognitive impairment in the study by Havins et al. [65]. Third, the dimension of dysphoria comprised ten items associated with dysphoric mood and dissatisfaction with life (e.g., *Do you feel that your life is empty*?). Fourth, the dimension of vigor/withdrawal comprised ten items related to the level of working energy (physical, emotional, and cognitive liveliness), and emotional difficulties or withdrawal of social interaction (e.g., *Do you prefer to stay at home, rather than going out and doing new things?*). Finally, following the result of factor analysis by Haavisto and Boron [42], GDS20 *“Is it hard for you to get started on new projects?”*, having a large factor loading to the dimensions of vigor/withdrawal and cognitive concerns, was included in both dimensions for DIF analysis because the lordif can be run if there are at least four items in a dimension.

### RQ2. Item quality and reliability of GDS

This study revealed that all GDS items contributed to the meaningful measurement and afforded a well-assessed depression level in the elderly, except for GDS23 in the dimension of dysphoria. It is because GDS 23 revealed a misfit value of the outfit MNSQ. A fit value of more than 1 signifies more variation in the data than the expected Rasch model (data underfit) [48, 50]. Among the items of dysphoria, it is interesting to note that only GDS23 assesses dysphoric mood by comparing oneself to other people (*Do you think that most people are better off than you are?*). This item was assumed to be responded diversely by respondents, resulting in noise in the data. Boone et al. stated misfitting items can degrade the quality of measurement [50]. Additionally, Linacre claimed that the outfit is more sensitive to outliers/some respondents are strange in some way such as guessing or thoughtless errors [66]. As a result, Boone et al. suggested that participants that contribute to noise in the data should be excluded from analysis [50]. Nonetheless, if the item outfit is too large (i.e. GDS23), Linacre suggested only reporting infit may be appropriate [66]. Thus, some researchers were more concerned with the deviation in infit instead in the outfit value [48]. In addition, the general practice is to keep this item and observe how respondents react to it in future samples to maximize the validity [66].

With regard to the reliability of GDS, good EAP/PV reliability showed a set of item difficulty that would be stable if it was administered to other respondents with the same performance [38]. In fact, the items on cognitive concerns were not reliable when tested in Chinese elderly, consequently influencing the value of combined data [55]. It has been argued that reducing the number of items might decrease the reliability value [49]. This could be the rationale for this study, since the dimension of cognitive concerns comprised three items. Neumann, Neumann and Nehm added that the reliability might not essentially decrease if the dimension has high-quality items [49]. Thus, revision of items is recommended [66]. Additionally, person separation reliability was reported to be excellent, indicating that both Korean and Chinese respondents have excellent consistency in performance if given the same items with the same item difficulty. Nonetheless, Cronbach’s alpha results showed low reliability in the dimension of agitation and cognitive concerns. Person reliability increased for a larger sample size. Therefore, a larger number of respondents are expected to result in better reliability [50].

### RQ 3. Generalizability of GDS items on Chinese and Korean elderly

The study findings presented the item bias of GDS, showing different responses to each item of the depression scale by respondents from two different countries, which consequently impacted the performance of different items to assess the two comparable groups. Particularly, this study examined 18 country-related DIF items out of 28 GDS items. The following DIF analysis related issues were noted. First, this study demonstrated that parametric and non-parametric analyses yielded similar results, particularly, the same 14 DIF items in both analyses. Basokcu and Ogretmen also stated that parametric and non-parametric DIF analyses generally generated the same results in the final analysis [67]. The Mantel-Haenszel (non-parametric) was less powerful but was easier to use than logistic regression of IRT (parametric) because it did not need any specification of the model and was free from collinearity problems [56, 57]. As a matter of fact, four out of five non-uniform DIF detected in parametric were not revealed in the non-parametric analysis of this study, supporting the argument regarding the ill-evaluated and non-uniform DIF of the Mantel-Haenszel method [57]. Additionally, in the efficacy of testing non-uniform DIF, the parametric analysis also revealed the graphical application of DIF items. Studies have reported that after comparing various parametric methods, logistic regression has been reported to have the best balance of detecting power and controlling Type I error rate [56, 67]. Second, lordif analysis could be performed if there are at least four items in a dimension; thus, GDS20 was included in both dimensions of cognitive concerns and vigor/withdrawal with consideration of the big factor loading. As a result, GDS20 was exhibited as a DIF with a large effect size in both dimensions, although different values of *χ*^2^ and *R*^2^ were elicited. Additionally, the ICC plots of GDS20 in the two dimensions showed an identical pattern. In this study, GDS20 was correlated with the dimension of cognitive concerns and vigor/withdrawal, and the combination of other items with GDS20 when analyzing DIF did not elicit a significant impact on the interpretation of GDS20 itself.

Furthermore, only the DIF revealed from the parametric analysis is discussed. Although China and Korea are culturally close, this study demonstrated severe DIF with five DIFs having a large effect size. First, after accounting for DIF for scoring, the Korean elderly were expected to score higher at agitation across different levels of depression. This dimension consisted of two DIF items with a large effect size: GDS6 *“Are you bothered by thoughts you can’t get out of your head?”* and GDS29 *“Is it easy for you to make decisions?”* and one DIF item with moderate effect size *“GDS13: Do you frequently worry about the future?”.* These items are related to perceived feelings of worry, fear, or something burdensome in the past or future. It was reported that there is a large cultural difference between the two countries in terms of uncertainty avoidance, where Korea is more intolerant to ambiguous situations (see www.hofstede-insights.com). High uncertainty avoidance is more threatened by an unknown situation, which consequently prompts worry and social anxiety [68]. As shown in this study, this cultural factor impacts how Koreans who have the same level of worry as Chinese are likely to score higher in filling the self-assessment.

Second, cognitive concerns are one of the main factors affecting depressive symptoms and disorders [69]. Nonetheless, there have been diverse definitions related to the prevalence and incidence of mild cognitive impairment reported by different articles, which has led to the critical challenge of understanding the social burden of this state [70]. Most studies suggest that older age and less education are the main factors associated with mild cognitive impairment [71]. In Korea, it is generally associated with being older, having a lower educational level, and illiteracy [72–74]. In China, it was reported that older age, less education [75–77], being a woman, having a lower socioeconomic status [75, 78], and living in rural residences [75, 79] had an impact on cognitive impairment. In this study, the effect of cumulative DIF on cognitive concerns showed a non-uniform DIF pattern. At higher trait levels, Koreans were expected to score slightly lower. Thus, we focused on lower levels of cognitive concern where Korean elderly people are estimated to score higher. The Korean elderly compared themselves with society when they were responding to the items of cognitive concerns. Korea undergoes a rapid transition from aging to an aged society. Besides Japan, South Korea was mentioned as one of the fastest aging nations in Asia [74]. The ascending trend of the median age of South Koreans from 1950 to 2050 was reported [80], showing an older population than China [81]. It was found that one-fourth of Koreans over the age of 65 had mild cognitive impairment [82]. Accordingly, it was assumed that an estimated higher score of Korean elderly was the impact of observing the prevalence and incidence of this case in Korean society.

Third, the dysphoria dimension illustrates the non-uniform DIF pattern as the effect of accumulative DIF. A large gap was noted in the lower level of dysphoria, where Korean elderly people were estimated to score lower. Four DIF were elicited from ten items regarding dysphoric mood and dissatisfaction with life: GDS2 *“Have you dropped many of your activities and interests?”*, GDS10 *“Do you often feel helpless?”*, GDS23 *“Do you think that most people are better off than you are?”*, GDS25 *“Do you frequently feel like crying?”*. Masculinity-femininity culture impacts the explanation of an individual’s emotional adjustment and perceptions [83, 84]. Compared to China, Korea has a feminine culture (see www.hofstede-insights.com), indicating that society places a dominant value on the quality of life and caring for others. Masculine cultures such as China would sacrifice the family and leisure priority for their success, while the feminine culture was associated negatively with unpleasant emotions such as anger and sadness [85, 86]. Higher levels of depression were noted in masculine than in feminine culture [87]. Although Korean and Chinese are included as societies with a restrained culture (inattention to leisure time, indulging is wrong, and controlling the gratification of their desires), China shows a slightly higher score than Korea on restraint culture.

Fourth, the vigor/withdrawal dimension also showed a non-uniform DIF effect with a large gap at a higher level of trait. It was estimated that Koreans scored lower on vigor/withdrawal than the Chinese elderly. This dimension is related to working energy (physical, emotional, and cognitive liveliness) and emotional difficulties or withdrawal of social interaction. Social involvement is a crucial factor impacting dysphoric mood, as well as vigor/withdrawal [88, 89]. As reported, China and Korea are considered collectivist countries. However, China had a slightly higher score for individualism. The more individualist culture, the more self-directed view, autonomous, and independent from others in terms of feeling, behavior, and thought. Previous research has found that individualistic cultures experience more intense extraversion and neuroticism [83, 90, 91]. When eight DIF were accounted for scoring, the elderly in a more collectivist culture with the same level of trait estimated to score lower.

## Conclusion and implication

Due to the increase in the elderly population and those with depression, various depression related health issues have gotten attention. This study examined the validation of GDS focus on testing generalizability of items on different countries. The findings showed that the four-dimensional model of geriatric depression scale (GDS) was the best framework to measure the level of depression among Chinese and Korean elderly population. Specifically, the measured traits were categorized into agitation, cognitive concerns, dysphoria, and vigor/withdrawal. The quality of the GDS was presented by fit items in all dimensions, except for GDS23, indicating that almost all items contribute to measuring the intended trait. Additionally, excellent person separation reliability and acceptable item reliability were revealed, except for the cognitive concerns dimension. This study recognized the item bias of GDS, particularly in measuring different patterns of expected scores from the respondents with the same trait coming from two different countries. Although China and Korea are geographically and culturally close, severe DIF in this study implies that different cultural backgrounds impact how the elderly interpret particular items of GDS.

The current study results show that GDS, a representative depression measurement, has significant DIF when used in China and Korea and potentially warn that depression-related research may not be generalized. This issue probably leads to the distortion of the accurate estimation of individual scores and the optimal decision to treat individuals. As the importance of mental health care has emerged particularly since the start of the Coronavirus Disease-19 (COVID-19) pandemic, more international comparative studies related to depression are expected to be investigated. In this sense, this study may be worthwhile and can be implemented to other scales of depression.

DIF studies have long been conducted in clinical research. It is also true that studies on the biological and sociocultural background related to depression have been conducted. The current study highlighted to address the need for researchers and administrators to be more aware of irrelevant factors that might conflate in patients’ cognition when responding to the self-reported measures. To compare the influence of various factors on depression, studies to assess measurement equivalence is necessary. Additionally, this study recommends the DIF research in another clinical topics such as stress, anxiety, and insomnia by considering irrelevant factors (i.e., gender, socioeconomic status, language, etc.). Therefore, our further study design will concern the bias scores and how they are associated with socio-demographic information. Furthermore, DIF study also can be applied for more than two groups, considering more flexible methods for international comparison studies.

### Limitation

Although this study provided comprehensive validation procedures for the instrument, it did not address how to overcome the misfit item, lower reliability value, and how to solve the DIF item. In addition, DIF analysis only examine the item that functions differently to different group of respondents, but it did not examine the cause of DIF. The result of non-uniform DIF was only interpreted based on the large difference in the expected scores and the discussion of DIF was only based on previous literature reviews related to depression in China and Korea. Finally, this study did not explore qualitatively the occurrence of DIF item by conducting an interview to the salience participants (affected by DIF item significantly) to complement the cultural discussion.

## Supplementary Information

**Additional file 1.**

## Data Availability

The datasets used and/or analyzed during the current study are available from the corresponding author on reasonable request.

## Abbreviations

AIC: Akaike information criterion
COVID-19: Coronavirus disease-19
CTT: Classical test theory
DIF: Differential item functioning
EAP/PV: A posteriori/plausible value
GDS: Geriatric depression scale
IRT: Item response theory
MNSQ: Mean square values
TCC: Test characteristic curve
