# Supplementary material for: Item bias on the geriatric depression scale (GDS): investigating the quality and generalizability of GDS on Chinese and Korean community-dwelling elderly population
Source: BMC Geriatr. 2021 Nov 9;21:637. doi: 10.1186/s12877-021-02516-z (PMC8577178; doi:10.1186/s12877-021-02516-z)
Supplement: Supplementary file 1 — Additional file 1. [file 12877_2021_2516_MOESM1_ESM.docx]

**APPENDIX**

DIF detected in GDS by performing parametric analysis

| **Dimension** | **DIF** | **Type of DIF** | **DIF Magnitude** | **DIF-Free Item** |
| --- | --- | --- | --- | --- |
| Dimension 1: Agitation | GDS6: Are you bothered by thoughts you can’t get out of your head? | Both | Large | GDS8: Are you afraid that something bad is going to happen to you? |
|  | GDS13: Do you frequently worry about the future? | Uniform | Moderate | GDS18: Do you worry a lot about the past? |
|  | GDS29: Is it easy for you to make decisions? | Uniform | Large |  |
| Dimension 2: Cognitive concerns | GDS14: Do you feel you have more problems with memory than most? | Both | Large | GDS30: Is your mind as clear as it used to be? |
|  | GDS26: Do you have trouble concentrating? | Non-uniform | Moderate |  |
|  | GDS20: Is it hard for you to get started on new projects? | Both | Large |  |
| Dimension 3: Dysphoria | GDS2: Have you dropped many of your activities and interests? | Uniform | Negligible | GDS1: Are you basically satisfied with your life? |
|  | GDS10: Do you often feel helpless? | Uniform | Negligible | GDS3: Do you feel that your life is empty? |
|  | GDS23: Do you think that most people are better off than you are? | Uniform | Moderate | GDS4: Do you often get bored? |
|  | GDS25: Do you frequently feel like crying? | Non-uniform | Moderate | GDS11: Do you often get restless and fidgety? |
|  |  |  |  | GDS16: Do you often feel downhearted and blue? |
|  |  |  |  | GDS24: Do you frequently get upset over little things? |
| Dimension 4: Vigor/withdrawal | GDS5: Are you hopeful about the future? | Uniform | Moderate | GDS22: Do you feel that your situation is hopeless? |
|  | GDS12: Do you prefer to stay at home, rather than going out and doing new things? | Uniform | Moderate | GDS27: Do you enjoy getting up in the morning? |
|  | GDS15: Do you think it is wonderful to be alive now? | Non-uniform | Moderate |  |
|  | GDS17: Do you feel pretty worthless the way you are now? | Non-uniform | Moderate |  |
|  | GDS19: Do you find life very exciting? | Non-uniform | Negligible |  |
|  | GDS20: Is it hard for you to get started on new projects? | Uniform | Large |  |
|  | GDS21: Do you feel full of energy? | Uniform | Negligible |  |
|  | GDS28: Do you prefer to avoid social gatherings? | Both | Moderate |  |
